# A National Network of Safe Havens: Scottish Perspective

**DOI:** 10.2196/31684

**Published:** 2022-03-09

**Authors:** Chuang Gao, Mark McGilchrist, Shahzad Mumtaz, Christopher Hall, Lesley Ann Anderson, John Zurowski, Sharon Gordon, Joanne Lumsden, Vicky Munro, Artur Wozniak, Michael Sibley, Christopher Banks, Chris Duncan, Pamela Linksted, Alastair Hume, Catherine L Stables, Charlie Mayor, Jacqueline Caldwell, Katie Wilde, Christian Cole, Emily Jefferson

**Affiliations:** 1 Health Informatics Centre Ninewells Hospital & Medical School University of Dundee Dundee United Kingdom; 2 Centre for Health Data Science University of Aberdeen Aberdeen United Kingdom; 3 Imaging Centre of Excellence Queen Elizabeth University Hospital Glasgow United Kingdom; 4 Grampian Data Safe Haven Aberdeen Centre for Health Data Science University of Aberdeen Aberdeen United Kingdom; 5 Electronic Data Research and Innovation Service Public Health Scotland Edinburgh United Kingdom; 6 Lothian Research Safe Haven Department of Public Health and Health Policy National Health Service Lothian Edinburgh United Kingdom; 7 EPCC University of Edinburgh Edinburgh United Kingdom; 8 DataLoch Usher Institute University of Edinburgh Edinburgh United Kingdom; 9 Glasgow Safe Haven Research and Development division of National Health Service Greater Glasgow and Clyde Glasgow United Kingdom

**Keywords:** electronic health records, Safe Haven, data governance

## Abstract

For over a decade, Scotland has implemented and operationalized a system of Safe Havens, which provides secure analytics platforms for researchers to access linked, deidentified electronic health records (EHRs) while managing the risk of unauthorized reidentification. In this paper, a perspective is provided on the state-of-the-art Scottish Safe Haven network, including its evolution, to define the key activities required to scale the Scottish Safe Haven network’s capability to facilitate research and health care improvement initiatives. A set of processes related to EHR data and their delivery in Scotland have been discussed. An interview with each Safe Haven was conducted to understand their services in detail, as well as their commonalities. The results show how Safe Havens in Scotland have protected privacy while facilitating the reuse of the EHR data. This study provides a common definition of a Safe Haven and promotes a consistent understanding among the Scottish Safe Haven network and the clinical and academic research community. We conclude by identifying areas where efficiencies across the network can be made to meet the needs of population-level studies at scale.

## Introduction

### Background

Electronic health records (EHRs) are routinely collected data that are generated when an individual receives care in a health care setting. EHRs typically contain records of medical history, diagnoses, medications, allergies, immunizations, other treatments, and laboratory results [1]. The records can be generated in different settings (eg, primary care facilities, such as clinics and health care centers, and secondary care facilities, such as hospitals and emergency care centers). Although the primary purpose of EHRs is to improve the direct care of patients, they also have some other purposes that are termed *secondary use* or *reuse* [2]. Using EHR data in research is one such type of secondary use [3,4].

Safe Havens are secure environments that have been widely used to support access to EHRs for research while protecting patient identity and privacy [5,6]. The 4 Safe Havens collaborating as part of the UK-wide Farr Institute were described by Lea et al [5] and were found to have different processes, controls, and environments. In Scotland, a network of 5 Safe Havens has been established to support EHR reuse and, over the past decade, has enabled researchers to access data at scale [6].

The Scottish network of Safe Havens has been highly successful in supporting research. Over the past 5 years, the network has supported >1000 research studies. There are a small number of research and innovation projects (eg, the Industrial Center for Artificial Intelligence Research in Digital diagnostics [7] and Research Data Scotland [8]) that are collaborations across Safe Havens. However, most research projects are delivered by a single Safe Haven. Each Safe Haven maintains and controls access to EHR data collected from their geographically local regions and therefore has detailed knowledge of these data sets. The exception in Scotland is the national Safe Haven (electronic Data Research and Innovation Service [eDRIS]), which holds national-level data sets. Researchers generally only access either the breadth of the nationally held data, with high cohort coverage, which are collected at a Scottish level, or the depth of the local clinical data, which has more detailed information about persons or entities from the regional Safe Havens.

Representatives from each Safe Haven within the network meet regularly and are supported and chaired by the Scottish government’s Chief Scientist Office. The Safe Havens collaborate to develop and share best practices. The network is primarily funded on a cost-recovery basis by charging researchers for services, with some Safe Havens also receiving some core support from the National Health Service (NHS) Scotland Research and Development funds.

This study provides an analysis of the infrastructure, operations, and features of each Safe Haven and assesses how these affect the interoperability and technical options to support multi–Safe Haven projects. We present how Safe Havens in Scotland have protected privacy and facilitated the reuse of the EHR data.

### What Is a Safe Haven?

#### Overview

Safe Havens have evolved as a set of processes for supporting researchers accessing sensitive data in a streamlined and secure way while maintaining patient confidentiality [5,9,10]. The term *Safe* *Haven* is widely used but can have different meanings to different people and in different contexts. Barton et al [11] described, in detail, the origins and evolution of the term. A Safe Haven was defined as follows:

A repository in which useful but potentially sensitive data may be kept securely under governance and informatics systems that are fit-for-purpose and appropriately tailored to the nature of the data being maintained, and may be accessed and utilised by legitimate users undertaking work and research contributing to biomedicine, health and/or to the ongoing development of healthcare systems.

Concerning health data, other Safe Havens or similar infrastructures [5,11] exist nationally [12-14] and internationally [15-17]. Kavianpour et al [18] provided a review of trusted research environments based on the interviews of 20 UK national and international Safe Havens. This paper provides a perspective on Safe Havens in Scotland and is based upon the direct experiences of the authors.

**Figure 1:**
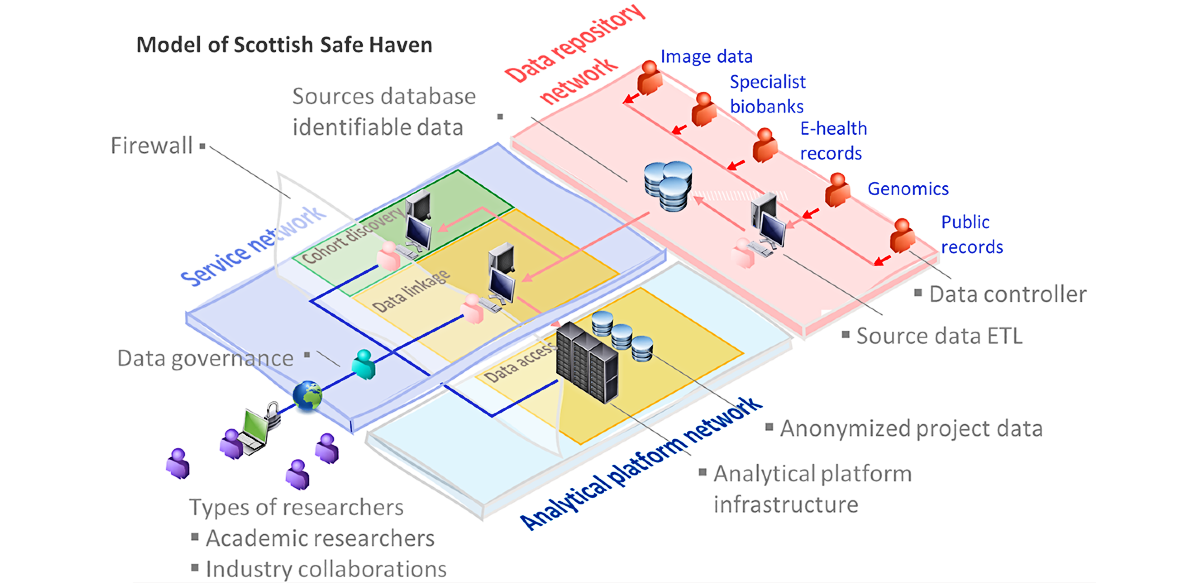
Model of Scottish Safe Havens. Researchers have access to the Safe Haven application process after data governance approvals. Safe Haven staff link and deidentify data and make them available in the analytic platform for researchers to analyze. ETL: extract, transform, and load.

Figure 1 provides a model of how Scottish Safe Havens are structured. We have identified that Scottish Safe Havens mainly offer services that are described in the following sections.

#### A Data Processor and Data Repository Management

This involves the secure handling and linking of data from multiple sources and possible hosting or managing of longitudinal data (detailed information about persons or entities, such as conditions, hospital admissions, and prescription data). Scottish Safe Havens can also provide the function of a *trusted third party* [6,19]. They can support the linkage of identifiable information where the roles of *indexer* and *linker* (see detailed definition in *Data Linkage* section) are separated so that no single organization or individual has visibility of another organization’s identifiable data linked to their descriptive data [20,21]. Safe Havens function as data processors [6] for any given data set and agree on terms with each data controller (Safe Havens can also be the data controller) to ensure that activities are centrally logged, monitored, and audited [6].

#### Analytical Platform

An analytical platform is a highly secure, high-performance computing environment that enables researchers to securely analyze data without the row-level deidentified data leaving the environment (only aggregate level results can be exported). Strict governance and controls are implemented to ensure data security in the analytical platform.

#### Research Support

The Safe Haven coordinators provide support to researchers navigating the data requirements and permissions landscape and provide a review mechanism to share the lessons from one project to the next. Some Scottish Safe Havens provide support for analysis. Internal Safe Haven data scientists can help the research group with statistical analysis.

The term *Safe Haven* is defined here as the overarching service that combines the previously mentioned services: a data processor and data repository, an analytics platform, and research support.

The Scottish Safe Havens follow the *five safes* principles of a trusted research environment—safe people, safe project, safe setting, safe data, and safe output [14]—as described within the Health Data Research United Kingdom green paper [14].

### Scottish Federated Network of Safe Havens

The network of 5 Safe Havens operating in Scotland is accredited by the Scottish government and each Safe Haven adheres to the Scottish Safe Haven Charter [6]. Each offers the 3 services, which are described in the *What is a Safe Haven*? section, with different data access procedures (subject to the necessary local governance approvals), applied to different data sources and with different standard operating procedures.

There are 4 regional Safe Havens and 1 national Safe Haven. There is a regional Safe Haven for each research and development node of the NHS supported by the Scottish government’s Chief Scientist Office [22]. They are provisioned by partnerships between the NHS boards within each research and development node and with a leading university from the region. Whether the primary contact organization for a Safe Haven is an NHS board or a university differs between regional Safe Havens (Table 1). eDRIS [23], part of Public Health Scotland (PHS) [24], commissions the Edinburgh Parallel Computing Centre (EPCC) [25], University of Edinburgh, to provide the national Safe Haven. Grampian Data Safe Haven (DaSH) [26], a collaboration between the University of Aberdeen and NHS Grampian, is the Safe Haven for the Grampian region encompassing Aberdeen City, Aberdeenshire, and Moray. The Health Informatics Center (HIC) [27] at the University of Dundee covers the Tayside and Fife regions. The Glasgow [28] and Lothian or DataLoch Safe Havens [29,30] are led by the NHS, covering the west of Scotland, Edinburgh, and the South East region, and working in collaboration with the Glasgow and Edinburgh Universities, respectively.

**Table 1:** A summary table of Safe Haven properties.

Function		Safe Haven				
		eDRIS^a^ (national)	DaSH^b^	Glasgow Safe Haven	HIC^c^	Lothian or DataLoch^d^
**General and data governance**						
	Safe Haven affiliation	PHS^e^	UoA^f^ or NHS^g^	NHS	UoD^h^ or NHS	NHS
	Analytical platform affiliation	UoE^i^ (EPCC^j^)	UoA	UoG^k^ (RCB^l^)	UoD	UoE (EPCC)
	Network for Safe Haven services (cohort building and linkage)	NHSnet or EPCC	NHSnet	NHSG^m^ or NHSnet	NHS	NHSL^n^ or NHSnet
	Network for analytical platform	UoE or Janet	UoA or Janet	UoG or Janet	UoD or Janet and secure public cloud	UoE or Janet
	Data repository network	NHSnet or EPCC	NHSnet	NHSnet	NHSnet	NHSnet
	Geographical region^o^	Scotland	NHS Grampian	West of Scotland	NHS Tayside and Fife	Lothian or South East of Scotland
	Population^p^	5.7 million	600,000	1.2 million	850,000	900,000
	Active projects in 2020	>600	>120	>100	>100	>20
	Controller or controllers	PHS+NRS^q^+Scottish government	Original data sources	Original data sources	Original data sources	Original data sources
	Processor or processors	eDRIS	DaSH	Glasgow Safe Haven	HIC	Lothian or DataLoch
	Governance committee	Health and Social Care PBPP^r^ and Statistics PBPP	North Node Privacy Advisory Committee	Privacy advisory committee	HIC governance committee	Data access committee
**Data discovery or metadata**						
	Feasibility	Manual or NDC^s^	Manual or local documents	Manual, local documents, or TriNetX^t^	Manual or using RDMP^u^ [19] automation	Manual or local documents
	Metadata provided with project extracts	No	Yes or standard (workflow)	Yes or bespoke	Yes or standard (RDMP)	Yes or bespoke
	Phenotype or cohort development	ICD^v^ code from user	By user	Locally stored algorithms or user	By user	By user or CALIBER Library
**Data linkage and dedeidentification**						
	Indexer	External (PHS for health data)	Internal	Internal	Internal (RDMP)	Internal
	Deidentification method	Workflow [31]	SQL procedure	Database views (usually SQL)	Workflow (RDMP)	SQL procedure
	CHI^w^ seeding	NSS^x^ or CHI Linkage Team	CHI Linkage Team or internal	Internal	Internal	CHI Linkage Team
**Analytic platform**						
	Archival	NHSnet and UoE (EPCC)	UoA	NHSnet and UoG (RCB)	NHSnet and UoD and secure public cloud	NHSnet and UoE (EPCC)
	Project data content standards	As source	As source or ICD	As source	As source	As source
	Project data format standards	CSV	SPSS, Stata, or CSV	CSV	CSV or database	CSV
**Data repository**						
	Data repository number	≥85	1	1	1	1 each
	Data repository ownership	No	Yes	Yes	Yes	Yes
	Source data metadata	NDC	Internal shared files	Internal shared files	RDMP	Data dictionaries
	Metadata publicly available	Yes	No	No	Yes	Yes^y^
	Number of data sets available	85	40	≥200	163	12
	Source data extract, transform, and load	Data management team PHS	Internal (SQL and Python)	Business Intelligence and Informatics in NHSG	Internal (RDMP)	Internal (SQL and Python)
	Repository uses CDM^z^	No (proprietary)	No (proprietary)	No (proprietary)	No (proprietary)	No (proprietary)

^a^eDRIS: electronic Data Research and Innovation Service.

^b^DaSH: Grampian Data Safe Haven.

^c^HIC: Health Informatics Centre.

^d^When this work was conducted, the Lothian Research Safe Haven (LRSH) and DataLoch were separate (though closely partnered). Since April 1, 2021, LRSH has been integrated within the DataLoch service.

^e^PHS: Public Health Scotland.

^f^UoA: University of Aberdeen.

^g^NHS: National Health Service.

^h^UoD: University of Dundee.

^i^UoE: University of Edinburgh.

^j^EPCC: Edinburgh Parallel Computing Centre.

^k^UoG: University of Glasgow.

^l^RCB: Robertson Centre For Biostatistics.

^m^NHSG: National Health Service Glasgow.

^n^NHSL: National Health Service Lothian.

^o^Regional Safe Havens have governance to request regional health board data. For example, Glasgow Safe Haven can request West of Scotland Health Board data.

^p^Safe Havens have access to historic records for patients who are deceased, which can increase the accessible data.

^q^NRS: National Records Scotland.

^r^PBPP: Public Benefit And Privacy Panel.

^s^NDC: national data catalog.

^t^TriNetX is a health research network tool that connects to assist drug discovery by helping pharmaceutical companies access clinical data. Glasgow Safe Haven has a TriNetX node. For data mapped into TriNetX tool, their study feasibility can be done using TriNetX.

^u^RDMP: Research Data Management Platform.

^v^ICD: International Statistical Classification of Diseases and Related Health Problems.

^w^CHI: community health index.

^x^NSS: National Services Scotland.

^y^COVID-19 data dictionary is on DataLoch website.

^z^CDM: common data model.

### Scottish NHS Data Sources

#### Overview

Scotland has a single health care provider (NHS Scotland) and world-leading national health–linked data assets from birth to death. In a high-level summary, the national Safe Haven has direct access to health administrative data, with high cohort coverage collected at a Scottish national level, and the regional Safe Havens have direct access to more detailed health data from clinical systems. Regional Safe Havens can work closely with local data custodians, which gives them easy access to additional data sources that are not routinely held (eg, other health data, educational data, or police data). Access to these other sources of data may require additional time because of different access processes and governance approvals.

The Research Data Scotland initiative [32] has been set up to streamline and support access to linked health and administrative data sets across the country.

#### National-Level NHS Data

The PHS collects national-level NHS [33] and administrative data to provide health information, health intelligence, statistical services, and advice to support the NHS in progressing quality improvement in health and care and facilitate robust planning and decision-making. These data sets can be accessed in the national Safe Haven. Each health board across Scotland provides a regular update of a subset of their identifiable administrative data to PHS. This is standardized by PHS to create homogeneous data within several national databases. Such data include Scottish Morbidity Records (SMRs) and community-dispensed prescriptions. SMR data cover several different data sets such as SMR00 (hospital outpatient), SMR01 (acute stay hospital admissions), SMR02 (maternity), SMR04 (psychiatric returns), SMR06 (cancer registry), SMR11 (neonatal), and SMR25 (substance misuse). National Records Scotland (NRS) records births, marriages, and deaths.

Prescription data are collected nationally in 2 different ways. Through the e-Pharmacy system [34], prescriptions written by general practitioners are captured directly in the system. The long-standing Data Capture Validation and Pricing paid system [35] is used in Scotland to capture dispensing data that determine remuneration for community pharmacies. The *watermarked* prescriptions go from general practitioners to the patient and then to a pharmacy and are then collected and transferred monthly to Data Capture Validation and Pricing or PHS for automated processing [36].

#### Regional-Level NHS Data

The regional Safe Havens all receive a subset of the data from the national standardized data sets (eg, SMR and prescribing data) from PHS, which includes only the patients who are residents or received health care within the relevant boards. They also have access to the deeply phenotyped data that are captured within local clinical systems but not collected at a national level. For example, they have access to the following data: microbiology, virology, laboratory test, stroke, and echocardiology. The type and level of available local data differ between Safe Havens. Individuals in Scotland are assigned a community health index (CHI) number [37] when they first interact with the health service. This is retained within their EHRs as much as possible throughout their health history. Regional Safe Havens use CHIs to link data sets to the nationally captured records for the population within their region.

#### Research Data

In addition to unconsented access to routinely collected administrative or clinical records, the national and regional Safe Havens can also host or manage researcher-collected consented data sets from many sources such as clinical trials and patient questionnaires. Compared with routinely collected EHRs, the research data often cover a narrower spectrum but provide more detailed information about the individual. Participants in research cohorts are volunteers who have consented to data access rules approved by ethics at the outset of the study. For example, Generation Scotland [38] is a resource of human biological samples and data that are available for medical research to create more effective treatments based on gene knowledge for the health, social, and economic benefit of Scotland and its people. Another example is the Scottish Health Research Register [39] cohort, in which >280,000 individuals consented and were recruited to allow for the genotyping of any remaining blood samples after routine tests and applied them to research on their health data [39].

Regional Safe Havens also host disease-specific study data [40-45]. The data within these studies can be collected from a range of sources: clinical data, patient surveys, and routinely collected EHR data.

Some disease registries were originally created at a regional level but were then rolled out nationally. For example, the Scottish Care Information–Diabetes (SCI-Diabetes) [46] disease registry was formed by curating and linking routinely collected data from the Tayside Region. It was later developed into a nationwide resource that now collects patient-reported outcome data. The data collected in SCI-Diabetes are used in clinical care. Extracts from SCI-Diabetes can also be linked on a study-by-study basis for research studies by the regional or the national Safe Havens.

Safe Havens can link research data to routinely collected administrative or clinical records and provide access to the combined data (in a deidentified form) within an analytical platform for analysis.

## Scottish Safe Havens

### Overview

Each Scottish Safe Haven has its data repository hosted on the NHS network (Table 1), except for the national Safe Haven, which also hosts some data within the EPCC on a secure university environment. Safe Havens have data-sharing agreements with multiple data controllers and regularly receive new data from them.

All Safe Havens have committed to an approach to data access based on analytical platforms. Each Safe Haven has either established or has access to an analytical platform.

There are differences among Safe Havens in how they achieve the 3 main services described. Table 1 summarizes each Scottish Safe Haven. In the following section, we discuss the Safe Havens in detail and their common deployment features.

### Data Governance and Workflow

The governance approval step looks into aspects of the project such as ethics, peer review, funding source, public benefit, and adherence to the *five safes*, as described in the *What is a Safe Haven?* section. The governance approval process varies among Safe Havens. Even for the same Safe Haven, different projects may require different governance approval processes to satisfy the different data controllers. However, the governance process for a standard deidentified project where data are accessed in an analytical platform is relatively streamlined. Each Safe Haven has a delegated governance authority or committee, as shown in Table 1, which is relatively fast and includes representatives from sponsors, ethics, lay members, and the NHS board to streamline the governance process. For the DaSH Safe Haven, projects with local researchers using local data can obtain governance approval through the North Node Privacy Advisory Committee process. The HIC Safe Haven’s *standard projects* (where deidentified data are analyzed by approved academic researchers within the analytical platform and the activity is funded by a peer-reviewed research grant) are covered by a blanket governance approval. The list of the supported standard projects is provided to the relevant governance committee for information rather than a request for approval for each one before the research commences. For Lothian or DataLoch, projects involving deidentified data within an analytical platform can obtain governance approval through their local data access committee process, which includes delegated Caldicott review.

Most Safe Haven responses to project requests adhere to a standard set of processes (eg, deidentified linked data are provided within an analytical platform for academic research). In exceptional circumstances, some projects require a different model, and such exceptions need to be justified to obtain governance approvals. Example exceptions include the prepared project data not being placed in an analytical platform and the project data having some identifiable information.

To work on identifiable EHR data within a data repository, Safe Haven staff members are either NHS employees or have honorary NHS contracts. All Safe Havens have rule-based segregation of the teams, specifying those with and without access to identifiable data. Only a handful of people in each Safe Haven can access the NHS network and see identifiable data. Other data sources (eg, administrative data generated by the government or research data generated by research institutions) can be linked to EHRs. The linkage is performed by the Safe Haven data linkage team, and the linked data sets are then hosted on the analytical platform for the approved researchers or investigators to access. At each stage, there is an oversight step to ensure all procedures are correctly followed and no unintentional identifiable data are released.

**Figure 2:**
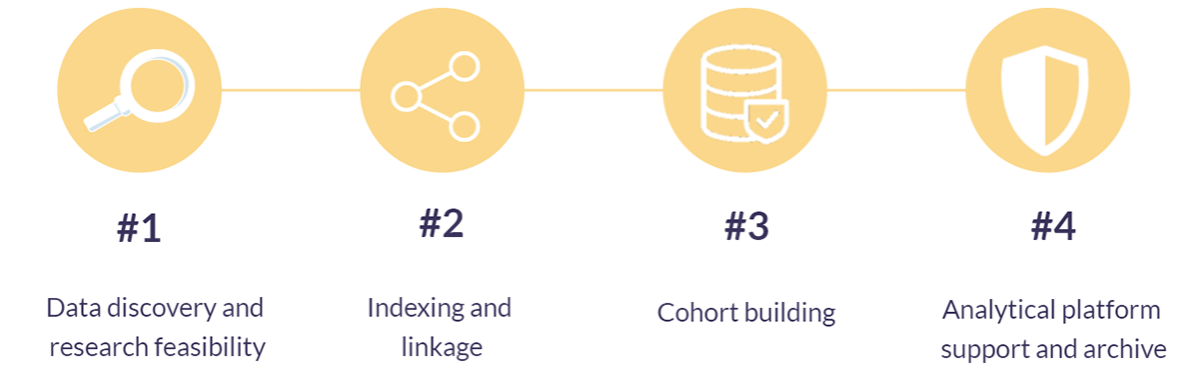
The Safe Haven project workflow describes the stages a Safe Haven takes to support a typical project. (1) Data discovery and research feasibility—users will initialize the application on the data governance aspects; (2) (optionally) index and link a research data set or administrative or clinical data set for hosting at a given analytic platform; (3) cohort building the selected or agreed data from Safe Haven data sets; (4) the transfer of extracted data to an analytic platform after the data governance has been checked; a user analyzes analytic platform data set. The project data set is archived at the end of the project.

The project workflow for a data request is consistent across the Safe Haven network, as shown in Figure 2. In the first step, the Safe Haven team runs research feasibility queries to identify the data needed for the research topic. Once funding and governance are in place, data linkage is conducted (as required). Data extracted from the NHS network are deidentified, validated, and assessed for disclosure before being released into the analytical platform. The details of linking and deidentification are given in the *Data Linkage* and *Data Deidentification* sections. The section *Data Formats in the Analytical Platform* provides discussions on the analytical platform support and data support. The archiving procedure of each Safe Haven and the infrastructures of each Safe Haven’s data repository are discussed in the *Data Repository Infrastructure* section.

### Data Discovery and Metadata

Research feasibility analysis and data discovery remain a manual process involving discussions between researchers and the Safe Haven teams. During the project planning stage, researchers contact the relevant Safe Haven by email or phone call. Data discovery and research feasibility are conducted by document exchange or a face-to-face meeting. Safe Havens in Scotland require a meeting to capture the requirements of each study and guide the governance process. Research feasibility is conducted by the Safe Haven by generating aggregate numbers for cohort or subcohort sizes based on the requirements defined by the researcher or researchers (eg, the number of people in the data with diabetes who are aged >65 years and who regularly have a prescription for insulin).

Researchers normally specify a phenotype or public phenotype algorithms to identify the correct cohort for their study. As shown in Table 1, no common standard procedure exists among the 5 Safe Havens to capture and reuse phenotype algorithms. However, DataLoch also uses the CALIBER phenotype library [47], whereas the Glasgow Safe Haven uses a suite of local matrix file storage phenotype algorithms, based on standard or published methods, which have been quality checked by clinicians. As the eDRIS mainly works on national data sets using the International Statistical Classification of Diseases and Related Health Problems (ICD) standard [48], it usually agrees on what the ICD codes are with the researcher and conducts the cohort building using the codes and any date or other constraints given by the researcher. The remaining Safe Havens—HIC and DaSH—normally rely on the researcher to define the cohort themselves, where researchers have the choice of phenotype definition (eg, CALIBER phenotypes or ICD codes). Cohort identification is sometimes an iterative process between researchers and the Safe Haven team where a data constraint is applied, the impact on cohort size is observed, and the constraint is adjusted to optimize the cohort.

At the national level, PHS produces a national data catalog [49] as a single definitive resource of information on Scottish health and social care data sets to assist cohort discovery.

Metadata provides the semantics associated with the Safe Haven data sets. There is limited visibility of the metadata and data provenance available from regional Safe Havens. Most of the Safe Havens list the names of their easily accessible databases on the web [24,26,28-30,49,50] and provide researchers with a brief overview of the most commonly accessed data sets. Both HIC and eDRIS add their metadata and data sets to the Health Data Research Innovation Gateway [51]. There is no common structure in EHR data storage across the health care system in Scotland. As only a limited number of data scientists and analysts have experience in handling NHS data, this lack of visibility of metadata and data provenance can lead to a gap in understanding by data scientists and analysts about what data are available. Some Safe Havens will only release detailed metadata once they have an initial understanding of the project’s needs. There are multiple initiatives, both internal [7,52] and external [8,51], that aim to improve the metadata visibility within the Scottish network of Safe Havens.

Research projects benefit from having clinical investigators who are familiar with NHS data or data scientists and analysts who have previous experience in working on Safe Haven projects within their project team. Such individuals can help identify what data are available and advise and support the data scientists and analysts working on the project. Most Safe Haven projects generally require a suitable sponsor with relevant expertise to take responsibility for the initiation and management of the project and support the project as an ethical safeguard.

All 5 Safe Havens provide research projects with metadata at the field level once a project is funded and approved and data extracts are provided for analysis. However, feasibility discussions will generally take place at the *conceptual* level. For example, a cohort definition may involve a fasting glucose constraint. The Safe Haven team will confirm that such a constraint is possible without disclosing the precise fields. To avoid bias and to get researchers to articulate what they need and what is available, this *conceptual*-level feasibility can be quite limiting. The same could be true of the data extract requested (eg, delivering BMI rather than height and weight). However, data extraction and delivery are generally at the field level, and field-level metadata are provided to ensure researchers can perform their analyses.

Table 1 shows that eDRIS provides metadata details on its website metadata (similar to the Cribsheet [53] on SMR), which can be used by researchers to define the fields they need when applying for data access. Researchers using regional Safe Havens can also use this metadata information for the nationally standardized data sets that the regional Safe Havens hold for the subset of their region (eg, SMR data). None of the Safe Havens provide non–field-based metadata, such as through an ontology.

The eDRIS Safe Haven does not provide bespoke metadata to the user when delivering the project data. DaSH and HIC Safe Havens have a standard workflow and delivery format for supplying project-specific metadata to each project. In the Glasgow and Lothian or Dataloch Safe Havens, projects are provided with all available metadata and provenance information. No standard format is used, and thus, the included information varies from project to project.

Safe Havens have different approaches to storing metadata about data sets in their data repositories. For eDRIS, PHS updates and maintains the national data catalog that contains all the metadata for national data sets. The HIC Safe Haven uses an in-house, open-source software tool called the Research Data Management Platform (RDMP) [19] for importing data to their servers. The RDMP generates consistently formatted metadata for imported data sets. The Lothian or DataLoch Safe Haven provides *data dictionaries*, which include metadata, for all the data sets in their Relational Database Management System (RDBMS). The Glasgow Safe Haven and the DaSH Safe Haven have internal document spaces to host the metadata and provenance provided by various data sources, which are manually entered and updated by staff. The lack of standard procedures in the Scottish Safe Haven network has resulted in the available metadata varying among data sets. Highly processed data sets—which have gone through extract, transform, and load (ETL) procedures—have field parameters and rules imposed on them. These data sets have rich metadata associated with them. However, most clinical data are inherently of variable quality, with poor coverage and inconsistent and missing fields. The data set metadata do not typically inform the user of the data variability or quality issues in the original data.

### Data Linkage

#### Overview

**Figure 3:**
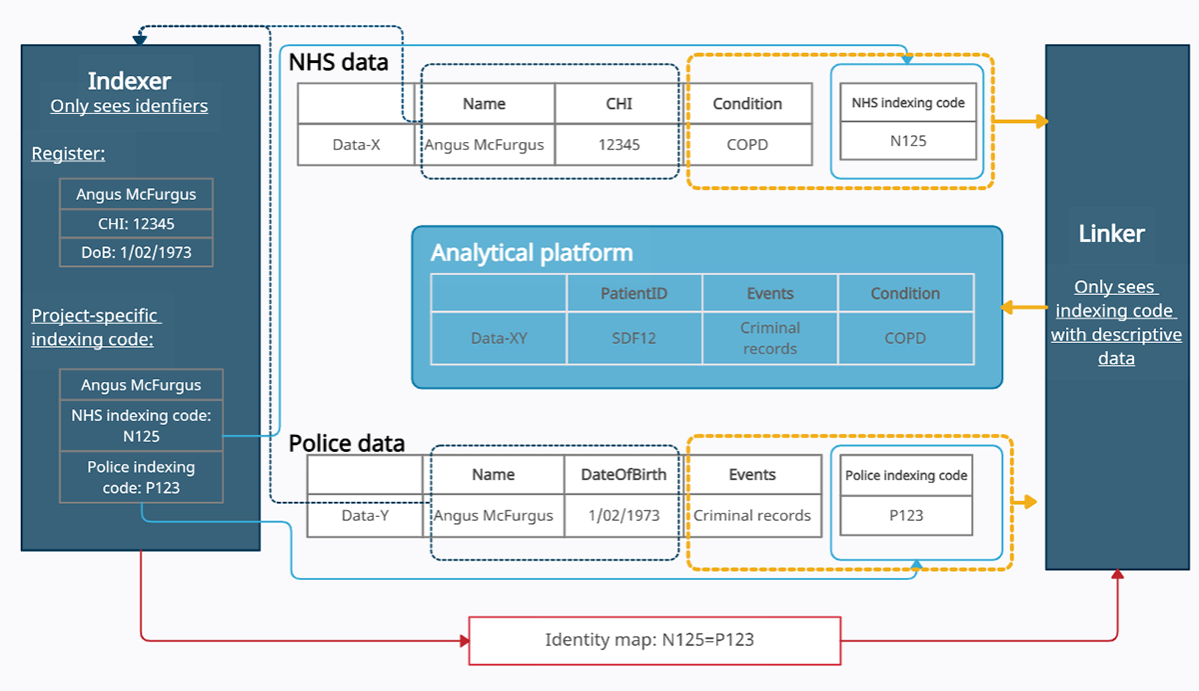
Data indexing and linking services in Scotland. CHI: community health index; COPD: chronic obstructive pulmonary disease; NHS: National Health Service.

To answer many research questions, data linkage is required to enrich information about a defined cohort. Some Safe Haven projects involve linking NHS data with non-NHS data. Figure 3 illustrates the indexing and linking services in the Scotland Safe Haven network.

According to the guiding principles of data linkage [21], an indexer is defined as follows:

Individual (or body) who receives personal data from one or more Data Controllers and determines which records in each dataset relate to the same individual (or entity). The indexer creates a unique reference for each individual (or entity) and a corresponding key to allow the data from the different sources to be joined.

Thus, an indexing service [21] returns a unique identifier for each individual, given an input data set of identifying information (eg, name, address, date of birth, and other operational identifiers such as CHI number). The relationship between the identifiers associated with multiple data sets is maintained by the indexing service. The indexing service does not have visibility of the descriptive data pertaining to any individual (also termed payload data, eg, an individual’s hospital admission information).

A linker or linking service is defined as follows: “Individual (or body) who receives datasets from data controllers and links them together using a key created by the indexer” [21].

In this way, only output identifiers from the indexer service are exposed to the linker; only the linking service and the researchers see the linked data [20].

In Scotland, the NHS maintains the CHI. This is a patient identifier that concatenates a unique number, the person’s date of birth, and their sex. CHI numbers are allocated at birth or on the first contact with the NHS in Scotland [37]. Linking health data to other data where both data sets already contain CHI numbers is straightforward. All 4 regional Safe Havens do this when preparing data for placement in an analytical platform using either software tools (eg, HIC use RDMP [19]) or RDBMS user interfaces.

The national Safe Haven, eDRIS, has established a data indexing and linkage procedure [31]. The input identifiers are personal identifiers, and the output identifier is an anonymized ID. eDRIS only receives data from providers with anonymized IDs and acts as a linker, placing the integrated data into a secure environment.

#### CHI Seeding

When linking to a source data set that does not have CHI numbers but features other identifiers, the indexing team will use *probabilistic matching* against the population spine. Related to the CHI, the population spine [31] contains the personal identifiers of all individuals in Scotland who have been in contact with NHS Scotland. The process of matching source data sets to the population spine is known as *CHI seeding*. The recent seeding of regional social care systems with CHI is an example of this. CHI seeding is also important for historical data analysis of EHRs before the introduction of CHI indexing. In Scotland, 2 teams provide national-level CHI seeding using probability matching: the NRS Indexing Team and the PHS CHI Linkage Team (CHILI). When a research project only needs NHS data, indexing would be conducted by CHILI.

Both eDRIS and the Lothian Safe Havens rely on the NRS or CHILI for CHI seeding. DaSH Safe Haven provides CHI seeding through the NRS Scotland’s indexing team; they will only do it themselves when they have specific personal identifiers available, such as patient name, and the data set comprises only a small amount of local Grampian patient records (approximately 500 people). Glasgow and HIC Safe Havens have a more established probabilistic matching routine developed and normally perform CHI seeding themselves. HIC has worked with local authorities to CHI seed their nonhealth data to be able to link it to health data.

### Data Deidentification

Deidentification is undertaken before a Safe Haven provides the data to an analytical platform for the researcher to access. Deidentification replaces information that could identify an individual in a data set with a study identifier (ID) for that individual, which is specific to that study, or dilutes the identifier to remove its individual nature. In linked, deidentified data sets, the study ID is the same across data sources, enabling researchers to link these data sources and understand which data correspond to the same individual within that study but without knowing their identity. This also means that deidentified IDs are unique to that project, and therefore, the same individual will have different IDs in different projects.

In general, Safe Havens apply consistent rules to identifiable data fields. Customizing deidentification rules based on the bespoke project requirements, governance approvals, and the variety of data sets can be accommodated. The treatment of identifiers depends on the project’s specific justification following data minimization principles [21]. For example, a date of birth can be processed to the first day of the month or be replaced with *age-at*, or it can be removed if it is not considered necessary for the analysis. A postcode can be replaced with a deprivation score or a Scottish Index of Multiple Deprivation rank [54], or it can be removed from the data. For biometric data, where, for example, the weight and height of the individual are included, Safe Havens often put such values into ranges. Each Safe Haven follows standard operating procedures for reproducibility, consistency, and error reduction. The Scottish Safe Havens are data controllers under the European Union provisions of the General Data Protection Regulation and are individually responsible for their local data. The Scottish Safe Havens are accredited by the Scottish Government and International Organization for Standardization 27001 [55,56] on the common information security standard. The Scottish Safe Haven network has not adopted a cross-network risk of reidentification metrics [57-59]. The *five safes* principle [14]—safe people, safe project, safe setting, safe data, and safe output—ensures that the risk of reidentification is very low.

All Safe Havens indicated that they find it challenging to deidentify clinical reports and other documents containing free text, which often contain personal identifiers such as phone numbers and names. Safe Havens often exclude entire fields from research extracts when they are not confident that such fields are safe to release. The Industrial Center for Artificial Intelligence Research in Digital diagnostics [7] uses *hidden in plain sight* techniques for identifiable data on images. eDRIS has developed algorithms to remove personal identifying information from the dose instructions on the Prescribing Information System (these can also extract structured information such as dose unit and frequency). As part of the Scottish Medical Imaging service [60] and PICTURES [61], there is work in progress to deidentify and create metadata from the text written by radiologists on their findings. This uses natural language processing and the CogStack framework [62].

### Data Formats in the Analytic Platform

In general, Safe Havens make few changes to the source data provided to researchers, these changes being limited to the process of deidentification. For example, there has been no attempt to harmonize data through the transformation of diagnosis codes or drug codes, where significant versioning occurs in longitudinal data. However, some Safe Havens do add derived data to data sets. Within HIC, for example, these data derivations and transformations can be applied either within the Safe Haven or at the point of extracting a deidentified research data set. This is done using RDMP, an open-source solution that allows custom coding, or a researcher-created statistics package code to be executed in a repeatable and reproducible manner. When requested by the researcher, DaSH Safe Haven can provide the Charlson Comorbidity Index [63] and Tonelli codes [64] alongside the ICD codes. Although data standards are not applied at data extraction and delivered to an analytical platform, standards are enforced for nationally captured data sets. A team in PHS works with the health boards and system suppliers to ensure the use of standards (eg, SMR data must be structured in an agreed-upon way and use agreed-upon coding systems for content).

Safe Havens make their best efforts to accommodate the requirements of projects. However, the software available in most analytical platforms is limited (Microsoft Office packages, SPSS (IBM Corp) [65], Stata (StataCorp) [66], SAS (SAS Institute, Inc) [67], and R (R Foundation for Statistical Computing) [68]), and thus, the output data formats are also limited to R, Excel, SPSS, or Stata files. The exception is the recently launched HIC hybrid, cloud-based, scalable analytical platform. This also includes the capability for software development, machine learning, and artificial intelligence development, including Python [69], Matrix Laboratory [70], and a suite of tools within Jupyter Notebooks (Sagemaker instance) [71] such as TensorFlow. The environment is also being enhanced to support multi-omic data [72] analysis through pipelines, using tools such as Plink [73] and Nexflow [74] with resource scheduling through Amazon Web Services Batch [75]. The analytical platform provides graphics processing units and high-performance computing capabilities.

For larger projects, where the number of rows is too high to manage in other formats, the HIC Safe Haven provides the data in an RDBMS in the analytical platform for use by researchers.

Researchers rely upon Safe Havens to archive the raw data and derived data products from their analysis as they are not permitted to export any of that data from an analytical platform. A research project may be archived for a period of between 5 and 30 years, depending on regulations and researcher or funder requirements. Archiving typically takes place using the analytical platform infrastructure. There can be significant costs for storing and securing large amounts of data, and a policy for long-term archival is being jointly developed by the Scottish Safe Havens.

### Data Repository Infrastructure

Safe Havens have their source EHRs on the NHS network, which are transferred to the service network (where the cohort building and linkage take place; Figure 1) when required. They create cohorts and associated data on the NHS infrastructure before the data go through the Safe Haven functions of linkage, deidentification, and transfer to analytical platform or platforms for researchers to access. The exception is eDRIS, which has some data sets managed securely in a university environment by EPCC.

**Figure 4:**
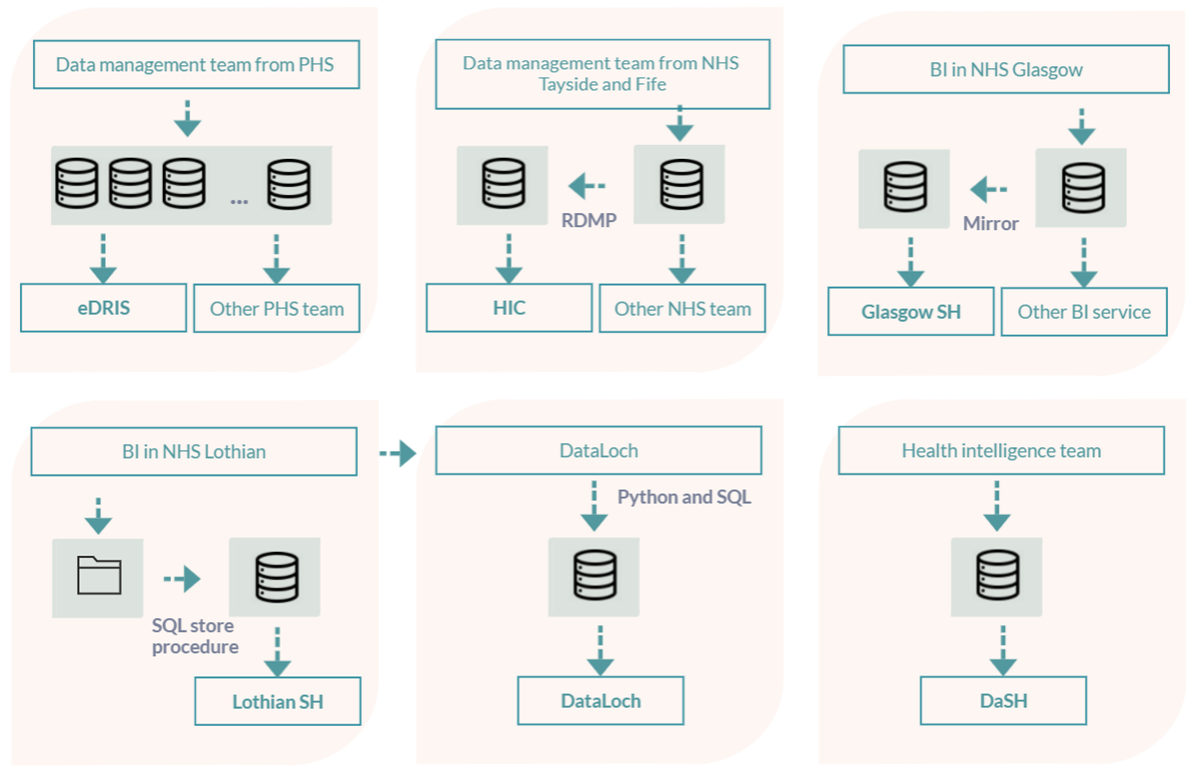
Safe Haven data repository networks. Upper row from left to right: electronic Data Research and Innovation Service, Health Informatics Center, and Glasgow Safe Haven. The lower row from left to right: Lothian, DataLoch, and Grampian Data Safe Haven Safe Haven. BI: Business Intelligence and Informatics; DaSH: Grampian Data Safe Haven; eDRIS: electronic Data Research and Innovation Service; HIC: Health Informatics Center; NHS: National Health Service; PHS: Public Health Scotland; RDMP: Research Data Management Platform; SH: Safe Haven.

The infrastructure and the ETL process for those data repositories vary among Safe Havens (Figure 4).

As shown in Table 1, eDRIS has access to 85 national NHS data sets; these are updated and maintained by PHS. There are data sets that eDRIS cannot access routinely; however, for a known cohort, they can request data from other teams within PHS. The data management team within PHS performs quality assurance after ETL using R or SPSS (in cases of legacy data). In addition to providing data to eDRIS, the data are also used to run hundreds of different reports and publications by other teams within PHS.

HIC’s data repository infrastructure and NHS Tayside or Fife data are colocated within the same data center. The HIC runs the University of Dundee–owned and managed servers connected to the NHS Tayside network and receives regular feeds of data from the NHS Tayside clinical systems and PHS (covering consented cohorts of research data and for the patients within the Tayside and Fife regions). The RDMP tool takes data from the feeds and performs ETL to clean and transform the data, which are then stored within structured databases.

Glasgow Safe Haven’s data repository mirrors some data from the routine data systems that are maintained by Business Intelligence and Informatics in NHS Glasgow. For custom NHS data or data collected for research projects (eg, some SMR, Prescribing Information System, all audit data, device data, and trial data), Glasgow Safe Haven staff will conduct the ETL themselves.

Lothian or DataLoch data repositories residing on the NHS Lothian information technology infrastructure use stored Python or SQL to load data updates from PHS and data feeds via Business Intelligence and Informatics within NHS Lothian for copies of data from local clinical systems.

NHS Grampian’s health intelligence team updates the DaSH Safe Haven repository monthly. Both Lothian or DataLoch and DaSH Safe Haven deal with changing data formats by separating new and old data.

## Discussion

### Principal Findings

#### Overview

Scotland has many strengths regarding enabling EHRs for reuse. There is a single NHS where patients are allocated CHI numbers that can be used to link their entire patient history. The Scottish network of Safe Havens has similar architectures, adheres to the Scottish Safe Haven Charter [6], is accredited by the Scottish Government and International Organization for Standardization 27001 [55,56], and is the common information security standard. Each regional Safe Haven has a rich and deep data source from their local health boards, and the national Safe Haven has the breadth of a whole-population view and close links to other health and social care data sources.

All the Safe Havens make use of two networks: (1) an analytical platform set up within university-managed networks and (2) data repositories set up mainly on NHS networks. The existing operational separation of source data repository, linkage infrastructure, and analytical platform provides a solid foundation for increasing collaborative work across national and multi–Safe Haven projects.

There are some barriers, as highlighted in the *Scottish Safe Haven* section, to making multi–Safe Haven projects as streamlined as possible. Addressing them in a coordinated manner would pave the way to achieving a federated system of Safe Havens in Scotland. These opportunities for improvement are described in the following sections.

#### Data Visibility

The depth of the Scottish data, which are hosted by regional Safe Havens (described in the *Scottish NHS Data Sources* section), is not widely used by the wider community. These data sets are unique to each regional Safe Haven and are difficult to bring up to a consistent national level. Interactions with researchers for feasibility, generating aggregate numbers, scoping projects, and providing quotes for work can be resource intensive. Many data requests to regional Safe Havens are from frequent users who know the specific data structures and terminologies used by each Safe Haven well. In making the regional data more visible and accessible, researchers will be better able to run projects using data from multiple Safe Havens.

#### Data Standards and Common Data Models

As shown in Table 1, the Safe Havens accept data that use any number of standards. Owing to the processing efficiency, the *create and destroy* model mandated by the Safe Haven Charter, and the fact that researchers normally prefer to have the original data, there has been little attempt to harmonize extracted data for placement in analytical platforms. If common data models such as Observational Medical Outcomes Partnership [76] and i2b2 star schema [77] were used, either for data repositories or analytical platforms, the burden on multi–Safe Haven projects would be reduced, and operational access to data would be faster and more predictable.

#### Governance

In the Safe Haven network, access to linked data is fragmented, with researchers and health care providers having to work with Safe Havens to obtain local, regional, or national data controllers’ approvals. Data governance, in general, is much easier at a local level. At the Scottish national level, application forms for submission to the Public Benefit and Privacy Panel for Health and Social Care and Statistics Public Benefit and Privacy Panel are normally required. This is a complex process and can take significant time for review and approval.

With the experience and knowledge gained from supporting projects requiring diverse local and regional data sets [78,79], and building capability for a federated network, we propose that the following aspects of the network be addressed in future research:

- The establishment of a shared method for cataloging and managing metadata would facilitate data discovery and research feasibility.
- To facilitate cross–Safe Haven data governance, standardization of the application interface specifications to Safe Havens would permit easier cross-access of Safe Havens by researchers.
- Health care delivery is explicitly devolved to local structures via health and social care partnerships in Scotland and associated legislation. With functions devolved to individual health boards, the linking of regional, available data will require greater collaboration across the organizations and appropriate benefit shares.

### Conclusions

The Safe Haven network in Scotland has supported over a thousand projects in the past 5 years, underpinning world-class research outputs. It not only brought grant research, jobs, and funding to Scotland but also enabled international health research with many countries such as Brazil and India.

This paper reports on the operational assessment of each of the 4 regional Safe Havens and the national Safe Haven. We compared a set of functions and services related to data forming part of EHRs in Scotland. We have described the operation of Scottish Safe Haven data services and functions and their technical implementation from the following points of view: (1) data governance and workflow, (2) data discovery and metadata, (3) data linkage, (4) data deidentification, (5) analytical platforms, and (6) data repository infrastructure. The results obtained should assist the Scottish Safe Havens to scale operations to larger cohorts and more diverse data, reduce timescales and operate more cost-effectively. More importantly, this work identified the responsibilities and work needed for each Scottish Safe Haven to contribute to the building of a national federated data-sharing platform. Although this paper has focused on experiences across Scotland, the findings will be of interest nationally or internationally to inform the understanding of the challenges that exist for the reuse of EHR data in clinical and other kinds of research.

## Abbreviations

CHI: community health index
CHILI: community health index Linkage Team
DaSH: Grampian Data Safe Haven
eDRIS: electronic Data Research and Innovation Service
EHR: electronic health record
EPCC: Edinburgh Parallel Computing Centre
ETL: extract, transform, and load
HIC: Health Informatics Center
ICD: International Statistical Classification of Diseases and Related Health Problems
NHS: National Health Service
NRS: National Records Scotland
PHS: Public Health Scotland
RDBMS: Relational Database Management System
RDMP: Research Data Management Platform
SCI-Diabetes: Scottish Care Information–Diabetes
SMR: Scottish Morbidity Record

## References

[1] World Health Organization (2016). From Innovation to Implementation: eHealth in the WHO European Region.

[2] Geissbuhler A, Safran C, Buchan I, Bellazzi R, Labkoff S, Eilenberg K, Leese A, Richardson C, Mantas J, Murray P, De Moor G (2013). Trustworthy reuse of health data: a transnational perspective. Int J Med Inform.

[3] Doiron D, Raina P, Fortier I, Linkage Between Cohorts and Health Care Utilization Data: Meeting of Canadian Stakeholders workshop participants (2013). Linking Canadian population health data: maximizing the potential of cohort and administrative data. Can J Public Health.

[4] A matter of life and death: how your health information can make a difference. AMRC.

[5] Lea NC, Nicholls J, Dobbs C, Sethi N, Cunningham J, Ainsworth J, Heaven M, Peacock T, Peacock A, Jones K, Laurie G, Kalra D (2016). Data safe havens and trust: toward a common understanding of trusted research platforms for governing secure and ethical health research. JMIR Med Inform.

[6] Charter for safe havens in Scotland: handling unconsented data from national health service patient records to support research and statistics. Scottish Government.

[7] iCAIRD.

[8] Research-Data-Scotland.

[9] Caldicott F (2013). Information: to share or not to share?. The Information Governance Toolkit.

[10] Thomas R, Walport M (2008). Data sharing review report. Data Sharing Review.

[11] Burton PR, Murtagh MJ, Boyd A, Williams JB, Dove ES, Wallace SE, Tassé AM, Little J, Chisholm RL, Gaye A, Hveem K, Brookes AJ, Goodwin P, Fistein J, Bobrow M, Knoppers BM (2015). Data Safe Havens in health research and healthcare. Bioinformatics.

[12] Jones KH, Ford DV, Thompson S, Lyons R (2019). A profile of the SAIL Databank on the UK Secure Research Platform. Int J Popul Data Sci.

[13] Jones KH, Ford DV, Ellwood-Thompson S, Lyons RA (2016). The UK Secure eResearch Platform for public health research: a case study. Lancet.

[14] Trusted Research Environments (TRE): a strategy to build public trust and meet changing health data science needs. HDRUK.

[15] Sciensano.

[16] Joseph K, Mahey J (2009). Validation of perinatal data in the Discharge Abstract Database of the Canadian Institute for Health Information. Chronic Dis Can.

[17] Schneider M, Radbone CG, Vasquez SA, Palfy M, Stanley AK (2019). Population data centre profile: SA NT DataLink (South Australia and Northern Territory). Int J Popul Data Sci.

[18] Kavianpour S, Sutherland J, Mansouri-Benssassi E, Coull N, Jefferson E (2021). A review of trusted research environments to support next generation capabilities based on interview analysis. JMIR Preprints.

[19] Nind T, Galloway J, McAllister G, Scobbie D, Bonney W, Hall C, Tramma L, Reel P, Groves M, Appleby P, Doney A, Guthrie B, Jefferson E (2018). The research data management platform (RDMP): a novel, process driven, open-source tool for the management of longitudinal cohorts of clinical data. Gigascience.

[20] Jefferson ER, Trucco﻿b E (2019). The challenges of assembling, maintaining and making available large data sets of clinical data for research. Computational Retinal Image Analysis.

[21] (2012). Joined-up data for better decisions: guiding principles for data linkage. Scottish Government.

[22] Chief Scientist Office.

[23] Products and Services. eDRIS.

[24] COVID-19 pandemic response. Public Health Scotland.

[25] EPCC.

[26] Grampian Population Platform: datasets permissioned for access for research purposes via DaSH. DaSH.

[27] Health Informatics Centre - trusted research environment. HIC.

[28] NHS-Greater-Glasgow-and-Clyde. Glasgow Safe Haven Services.

[29] Datasets. Lothian-Research-Safe-Haven.

[30] COVID-19 collaborative. DATALOCH.

[31] Data linkage. eDRIS.

[32] RDS - Research Data Scotland.

[33] National datasets. National Data Catalogue.

[34] ePharmacy user guide (Scotland). INPS.

[35] (2018). Glossary of terms: practice level prescribing data. Information Services Division, Scotland.

[36] Alvarez-Madrazo S, McTaggart S, Nangle C, Nicholson E, Bennie M (2016). Data resource profile: The Scottish National Prescribing Information System (PIS). Int J Epidemiol.

[37] CHI Number. ISD Scotland Data Dictionary.

[38] Generation Scotland.

[39] McKinstry B, Sullivan FM, Vasishta S, Armstrong R, Hanley J, Haughney J, Philip S, Smith BH, Wood A, Palmer CNA (2017). Cohort profile: the Scottish Research register SHARE. A register of people interested in research participation linked to NHS data sets. BMJ Open.

[40] EMBARC - The European Bronchiectasis Registry.

[41] DARTS (Diabetes Audit and Research in Tayside Scotland). GoDARTS.

[42] Childsmile – Improving the Oral Health of Children in Scotland.

[43] Sawhney S, Marks A, Fluck N, Levin A, Prescott G, Black C (2017). Intermediate and long-term outcomes of survivors of acute kidney injury episodes: a large population-based cohort study. Am J Kidney Dis.

[44] Ayorinde AA, Wilde K, Lemon J, Campbell D, Bhattacharya S (2016). Data resource profile: The Aberdeen Maternity and Neonatal Databank (AMND). Int J Epidemiol.

[45] Children of the 1950s. Aberdeen Birth Cohorts.

[46] SCI-Diabetes. Scottish Care Information.

[47] Denaxas S, Gonzalez-Izquierdo A, Direk K, Fitzpatrick NK, Fatemifar G, Banerjee A, Dobson RJ, Howe LJ, Kuan V, Lumbers RT, Pasea L, Patel RS, Shah AD, Hingorani AD, Sudlow C, Hemingway H (2019). UK phenomics platform for developing and validating electronic health record phenotypes: CALIBER. J Am Med Inform Assoc.

[48] International statistical classification of diseases and related health problems (ICD). World Health Organization.

[49] The National Data Catalogue (NDC). Public Health Scotland.

[50] Dataset inventory. HIC - University of Dundee.

[51] (2019). Specification for phase 2: technology partnership. Health Data Research Innovation Gateway, UK.

[52] Trust data provenance. Centre for Health Data Science.

[53] SMR crib sheets. Public Health Scotland.

[54] Scottish Index of Multiple Deprivation 2020. Scottish Government.

[55] Calder A, Watkins SG (2019). Information Security Risk Management for ISO 27001/ISO 27002, Third Edition.

[56] (2013). Information technology - Security techniques - Information security management systems - Requirements. International-Organization-for-Standardization, ISO/IEC 27001.

[57] SWEENEY L (2012). k-anonymity: a model for protecting privacy. Int J Uncertain Fuzz Knowl Based Syst.

[58] Machanavajjhala A, Kifer D, Gehrke J, Venkitasubramaniam M (2007). L-diversity: privacy beyond k-anonymity. ACM Trans Knowl Discov Data.

[59] Samarati P (2001). Protecting respondents identities in microdata release. IEEE Trans Knowl Data Eng.

[60] Scottish Medical Imaging (SMI) Service. Public Health Scotland.

[61] Nind T, Sutherland J, McAllister G, Hardy D, Hume A, MacLeod R, Caldwell J, Krueger S, Tramma L, Teviotdale R, Abdelatif M, Gillen K, Ward J, Scobbie D, Baillie I, Brooks A, Prodan B, Kerr W, Sloan-Murphy D, Herrera JF, McManus D, Morris C, Sinclair C, Baxter R, Parsons M, Morris A, Jefferson E (2020). An extensible big data software architecture managing a research resource of real-world clinical radiology data linked to other health data from the whole Scottish population. Gigascience.

[62] Jackson R, Kartoglu I, Stringer C, Gorrell G, Roberts A, Song X, Wu H, Agrawal A, Lui K, Groza T, Lewsley D, Northwood D, Folarin A, Stewart R, Dobson R (2018). CogStack - experiences of deploying integrated information retrieval and extraction services in a large National Health Service Foundation Trust hospital. BMC Med Inform Decis Mak.

[63] Sundararajan V, Henderson T, Perry C, Muggivan A, Quan H, Ghali WA (2004). New ICD-10 version of the Charlson comorbidity index predicted in-hospital mortality. J Clin Epidemiol.

[64] Tonelli M, Wiebe N, Fortin M, Guthrie B, Hemmelgarn BR, James MT, Klarenbach SW, Lewanczuk R, Manns BJ, Ronksley P, Sargious P, Straus S, Quan H, Alberta Kidney Disease Network (2015). Methods for identifying 30 chronic conditions: application to administrative data. BMC Med Inform Decis Mak.

[65] IBM SPSS Statistics. IBM.

[66] STATA.

[67] SAS - Analytics Software & Solutions.

[68] The R Project for Statistical Computing. The R Foundation.

[69] Python.

[70] MATLAB.

[71] Use Amazon SageMaker Notebook Instances. Amazon.

[72] Palsson B, Zengler K (2010). The challenges of integrating multi-omic data sets. Nat Chem Biol.

[73] Plink.

[74] Nextflow.

[75] AWS Batch. Amazon.

[76] OMOP Common Data Model. OHDSI.

[77] Murphy SN, Weber G, Mendis M, Gainer V, Chueh HC, Churchill S, Kohane I (2010). Serving the enterprise and beyond with informatics for integrating biology and the bedside (i2b2). J Am Med Inform Assoc.

[78] Shah AS, Anand A, Strachan FE, Ferry AV, Lee KK, Chapman AR, Sandeman D, Stables CL, Adamson PD, Andrews JP, Anwar MS, Hung J, Moss AJ, O'Brien R, Berry C, Findlay I, Walker S, Cruickshank A, Reid A, Gray A, Collinson PO, Apple FS, McAllister DA, Maguire D, Fox KA, Newby DE, Tuck C, Harkess R, Parker RA, Keerie C, Weir CJ, Mills NL, Marshall L, Stewart SD, Fujisawa T, Vallejos CA, Tsanas A, Hautvast M, McPherson J, McKinlay L, Malo J, Fischbacher CM, Croal BL, Leslie SJ, Walker A, Wackett T, Armstrong R, Stirling L, MacDonald C, Sadat I, Finlay F, Charles H, Linksted P, Young S, Alexander B, Duncan C (2018). High-sensitivity troponin in the evaluation of patients with suspected acute coronary syndrome: a stepped-wedge, cluster-randomised controlled trial. Lancet.

[79] No authors listed (2015). High sensitivity cardiac troponin and the under-diagnosis of myocardial infarction in women: prospective cohort study. Br Med J.

